# CP-25 exerts a protective effect against ConA-induced hepatitis *via* regulating inflammation and immune response

**DOI:** 10.3389/fphar.2022.1041671

**Published:** 2022-11-15

**Authors:** Nan Li, Jing-Jing Wu, Meng Qi, Zi-Ying Wang, Sheng-Nan Zhang, Xiu-Qin Li, Ting-Ting Chen, Mei-Fang Wang, Ling-Ling Zhang, Wei Wei, Wu-Yi Sun

**Affiliations:** ^1^ Institute of Clinical Pharmacology, Anhui Medical University, Key Laboratory of Anti-inflammatory and Immune Medicine, Ministry of Education, Anhui Collaborative Innovation Center of Anti-inflammatory and Immune Medicine, Hefei, China; ^2^ Department of Oncology, The Second Affiliated Hospital of Anhui Medical University, Hefei, China

**Keywords:** concanavalin A, CP-25, inflammation, hepatitis, inflammatory response

## Abstract

Hepatitis is a complex multifactorial pathological disorder, which can eventually lead to liver failure and even potentially be life threatening. Paeoniflorin-6′-O-benzene sulfonate (CP-25) has proven to have critical anti-inflammatory effects in arthritis. However, the effects of CP-25 in the pathogenesis of hepatitis remains unclear. In this experiment, mice were intragastrically administered with CP-25 (25, 50 and 100 mg/kg), and then ConA (25 mg/kg) was intravenous injected to establish hepatitis model *in vivo*. CP-25 administration attenuated liver damage and decreased ALT and AST activities in mice with hepatitis. Besides, CP-25 modulated immune responses including down-regulated the proportions of activated CD4^+^, activated CD8^+^ T cells, and ratio of Th1/Th2 in ConA-injected mice. Furthermore, ConA-mediated production of reactive oxygen species (ROS), release of inflammatory cytokines including IFN-γ, TNF-α, activation of MAPK pathways and nuclear translocation of nuclear factor-kappaB (NF-κB) were significantly decreased in CP-25 administrated mice. In ConA-stimulated RAW264.7 cells, CP-25 suppressed inflammatory cytokines secretion and reduced ROS level, which were consistent with animal experiments. Otherwise, the data showed that CP-25 restrained phosphorylation of ERK, JNK and p38 MAPK pathways influenced by ROS, accompanied with inhibiting NF-κB nuclear translocation. In conclusion, our findings indicated that CP-25 protected against ConA-induced hepatitis may through modulating immune responses and attenuating ROS-mediated inflammation via the MAPK/NF-κB signaling pathway.

## Introduction

Liver is the pivotal digestive organ and plays a crucial role in detoxification, metabolism, and immunity (Kubes and Jenne, 2018). Hepatitis caused by viral infections, drugs, food additives and alcohol can progress into cirrhosis, resulting in a significant health issue. It is reported that an inflammatory process would lead to hepatitis, which was characterized by excessive macrophages infiltration and propagation of inflammatory mediators (Jothimani et al., 2020). However, the precise mechanism of inflammatory regulation in hepatitis remains poorly understood.

Inflammation is considered to be an automatic defense response that benefits to maintain normal tissue function from potential harm caused by autoimmune damage or injury. But excessive accumulation of inflammatory cytokines could induce kidney, heart, lung and liver damage (Karki et al., 2021). Macrophages are the primary contributors to release amounts of inflammatory cytokines in response to liver inflammation and damage. The imbalance level of reactive oxygen species (ROS) in macrophages has evolved in liver tissue destruction, accompanying with oxidative stress and production of inflammatory cytokines (Zhang P et al., 2020). Concanavalin A (ConA)-induced hepatitis has been considered as a well-established experimental model due to its obvious pathological changes in inflammatory cytokines production and immune response (Rani et al., 2018). Multiple T cell subsets have been confirmed to be involved in the etiology of autoimmune disorders including hepatitis (Graham et al., 2021). We have previously shown that increased proportions of activated T cells and T helper (Th) cells contribute to liver injury in CCl_4_-induced fibrotic mice, and inhibiting the disorders of immune responses from various aspects could alleviate liver fibrosis (Sun J. C et al., 2020). Therefore, developing inflammatory and immunomodulatory agents could provide new prophylaxis or therapeutic approaches for hepatitis.

*Paeonia lactiflora* pall root exhibits multiple pharmacological activities, which has been an integral part of effective prescriptions for treatment of inflammatory and immune diseases. Paeoniflorin is the main bioactivity ingredient of total glycosides of paeony that presents in the root of *Paeonia lactiflora* (Zhang and Wei, 2020). Our lab has composed an innovative ester derivative of paeoniflorin named paeoniflorin-6′-O-benzene sulfonate (CP-25, patent number in China: ZL201210030616.4). CP-25 has superior intestinal absorption and improved lipid solubility compared with the paeoniflorin. Our lab has reported that CP-25 could reduce pro-inflammatory cytokines production in adjuvant-induced arthritis (Chang et al., 2016). Consistent with another study, CP-25 treatment showed a disease-attenuating effect on modulating T lymphocyte subsets in primary Sjögren’s syndrome mice (Gu et al., 2018). Our previous study presented that paeoniflorin displayed antifibrotic effects on liver fibrosis rats induced by porcine serum (Sun et al., 2012). Despite advanced evidence displayed the role of CP-25 in multiple diseases, it is still unclear whether it has an effect on ConA-induced hepatitis and the probable mechanisms.

In present experiments, ConA administration was used to establish hepatitis model. Our results displayed that CP-25 is not only as an immune response modulator in ConA-induced hepatitis, but restraining of inflammatory cytokines release may through down-regulating ROS influenced MAPK pathways activation and NF-κB nuclear translocation. Altogether, our findings indicated that CP-25 is a potential therapeutic agent in hepatitis.

## Materials and methods

### Animals and treatment

Male C57BL/6J mice (18 ± 22) g were purchased from the Animal Center of Anhui Medical University. The mice were kept in a temperature- and humidity-controlled room under standard 12-h light/dark cycles, maintained with administered food and water *ad libitum*. The Ethics Review Committee approved the animal experimental protocol at the Animal Experimentation of Anhui Medical University.

In this experiment, seven groups were divided at random from the mice: normal group, ConA-induced hepatitis model group, three dosages of CP-25-treated groups (25, 50, and 100 mg/kg), paeoniflorin-treated group (100 mg/kg), bicyclol-treated group (100 mg/kg). The effect of CP-25 and paeoniflorin at the same dose in the treatment of liver injury was compared. Meanwhile, bicyclol (Beijing Union Pharmaceutical Factory, Beijing, China) was served as a positive control. To establish the hepatitis mice model, mice received an intravenous injection of ConA (Solarbio, Beijing, China, C8110) at dose of 25 mg/kg. All drugs were diluted with 0.5% carboxymethyl cellulose sodium (CMC-Na) solution and intragastrically treated for 10 days before ConA injection. Other mice were intragastrically administered with an equivalent volume of CMC-Na solution. Then sacrificed the mice at indicated time point for liver, thymus, spleen and blood samples gathering.

### Cell culture and treatment

RAW264.7 cells were purchased from ATCC (Manassas, VA, United States), routinely maintained in DMEM (Gibco, CA, United States) supplemented with 10% fetal bovine serum (Zhejiang Tianhang Biotechnology Co., Ltd, Zhejiang, China) in a humidified incubator at 37°C in 5% CO_2_.

Several studies indicated that ConA administration obviously elevated inflammation in RAW264.7 cells (Zhang Z et al., 2020). Thus *in vitro*, RAW264.7 cells stimulated with ConA (10 μg/ml) was taken as model group, the cells treated by the same cell medium without ConA was taken as control group. The treatment groups were cultured with CP-25 (10^−7^–10^−5^ mol/L), meanwhile, ConA-stimulated group was treated with equal-volume DMEM. Then following experiments were conducted to verify the potential effect of CP-25.

### Ratio of liver, spleen and thymus weight to body weight

The liver, spleen and thymus were separated from the mice and immediately weighed. The liver, spleen and thymus indices were defined as: Liver, spleen or thymus index = liver, spleen or thymus weight/body weight × 100%.

### Liver histopathology

Mice liver tissues were collected and fixed with 4% formalin, the paraffin-embedded tissues cut into 4 µm sections. The prepared liver slices were stained by hematoxylin and eosin staining (H&E). Samples were photographed under a DFC7000 T color microscope camera (Leica Microsystems, Wetzlar, Germany) to display the pathological damage.

### Analysis of liver function

The serum was collected from blood samples by centrifugation at 3,000 r/min for 20 min and stored at -20 °C. The activities of serum alanine aminotransferase (ALT) and aspartate aminotransferase (AST) were detected by spectrophotometric assay kits (Nanjing Jiancheng Bioengineering Institute, Jiangsu, China, C009-two to one and C010-2–1), and showed as an international unit per litre (U/L).

### T cell viability assay

Thymus were aseptically separated from mice. Then, thymocyte suspensions were prepared by mechanical dissociation of the tissues. Thymocytes was re-suspended in a DMEM medium, and incubated with ConA in 96-well plates. 10 μl cell counting kit-8 (CCK-8) (Biosharp, Hefei, China, BS350A) reagents were supplied to each well after the incubation period. The absorbance at 450 nm was measured using an Infinite M1000 PRO microplate reader (Tecan Group Ltd., Männedorf, Switzerland).

### Preparation of spleen mononuclear cells and T cell subset analysis

The spleen tissue was removed and a single spleen suspension was harvested by mechanical separation of spleen through 70 μm filter and erythrocytes were lysed. Splenocytes were washed with PBS and re-suspended in DMEM medium. The antibody combinations CD3-PE (12-0038-42)/CD4-FITC (11-0041-81)/CD8-APC (17-0081-81) or CD4-FITC/CD8-APC/CD69-PE (12-0691-81) (eBioscience, San Diego, United States) were added into each tube, the expression of CD4 (CD8) was observed in CD3^+^ or CD69^+^ cell gate. Moreover, for intracellular IL-4 and IFN-γ staining, CD4-FITC antibody was used to stain surface marker, then samples were fixed, permeabilized to prevent nonspecific binding before staining with labelled IL-4-PE (12-7041-81)/IFN-γ-APC (17-7311-81) (eBioscience, San Diego, United States) antibodies. A single fluorescent dye sample was stained with CD3-PE, CD4-FITC, CD8-APC, CD69-PE, IL-4-PE and IFN-γ-APC respectively. Negative cells were stained without fluorescent dye. All the samples were tested using a CytoFLEX flow cytometry (Beckman, CA, United States), the data analysis was performed using a CytExpert software, and we have chosen the gate from comparing between negative and single fluorescent dye cells.

### Determination of cytokines by ELISA

Weighed fresh liver tissues were homogenized with PBS and centrifuged (4°C, 5,000×*g* for 5 min). *In vitro*, the cell supernatants in ConA-stimulated RAW264.7 cells were acquired. The levels of IFN-γ (ml063132), TNF-α (ml002095), IL-4 (ml002149) and IL-1β (ml063132) in all supernatants were measured by enzyme-linked immunosorbent assay (ELISA) kits (Shanghai Enzyme-linked Biotechnology Co., Ltd, Shanghai, China) according to the manufacturer’s instructions.

### Immunostaining and confocal microscopy

Liver sections were blocking in 3% BSA undertaken for 2 h to prevent nonspecific staining. After rinsing with PBS, sections were incubated with following primary and secondary antibodies: F4/80 (BioLegend, San Diego, CA, 123,101), Alexa Fluor 488 (Thermo Fisher, MA, United States, A11001). Negative control sections were incubated with PBS instead of primary antibody. Before imaging, sections should be counterstained with DAPI (Beyotime, Shanghai, China) to label the nuclei. Then tissue images were taken with a Leica TCS SP8 confocal microscope (Leica, Wetzlar, Germany).

### Determination of ROS production

ROS production in liver tissues and cells was detected by the oxidation of dihydroethidium (DHE, S0063) and 2′,7′-dichlorodihydrofluorescein diacetate (DCFH-DA, S0033S) (Beyotime Biotechnology, Shanghai, China). For DHE staining, liver cryosections were cultivated with 5 μmol/L DHE at the indicated temperature for 30 min and then photographed using DFC7000 T color microscope camera. Average staining intensities were quantified by ImageJ software (National Institutes of Health, MD, United States). *In vitro*, ConA-stimulated RAW264.7 cells were cultured with DCFH-DA and then analyzed on a CytoFLEX flow cytometer at excitation and emission wavelengths of 488 nm.

### Western blot analysis

Total proteins were harvested from liver tissues or RAW264.7 cells. Immunoblotting detection was conducted as previously described (Sun W. Y et al., 2020). The following antibodies were used: p-JNK (#9251S), JNK (#9252), p-ERK (#9101s), ERK (#9102), p-p38 (#9212s), and p38 (#9211s) (Cell Signaling Technology, Danvers, MA). Immunoblot band densitometry was quantified using ImageJ software. Three independent experiments were performed.

### Nuclear factor-kappaB (NF-κB) nuclear translocation assay

NF-κB p65 translocation of liver tissues was detected by immunohistochemical staining. The streptavidin/peroxidase method (Zhongshan Goldenbridge, LTD, Beijing, China, PV-9000) was used to detect the immunoreactivity. Each section was placed in 3% H_2_O_2_ in methanol and covered with anti-NF-κB p65 (Cell Signaling Technology, Danvers, MA, #8242). Then, peroxidase was visualized by incubation with Diaminobenzidine (DAPI, ZLI-9017) and counterstaining was done with hematoxylin. Slides were viewed through a DFC7000 T color microscope camera. Semiquantitative analysis was performed using ImageJ software.

RAW264.7 cells were incubated with ConA and then rinsed, fixed, permeabilized and incubated primary anti-NF-κB p65, followed by Alexa Fluor 555 (Thermo Fisher, MA, United States, A31572). Then counterstained with DAPI nuclear stain. Data of 1,000 cells per sample were acquired by an Amnis ImageStream X Mark II imaging flow cytometer (Amnis Corporation, Seattle, WA). IDEAS software was used to show brightfield images and obtain the proportions of NF-κB nuclear localization.

### Statistical analysis

All results were analyzed by SPSS software version 24.0 (SPSS Inc., Chicago, Illinois, United States), and data represented using GraphPad Prism Software 8.0 (San Diego, CA, United States). Values in figures are given as means ± SD if not otherwise indicated. Statistical significance was determined by one-way analysis of variance (ANOVA) for multiple comparisons. *p* < 0.05 was considered to be significant.

## Results

### CP-25 attenuated ConA-induced hepatitis

**FIGURE 1:**
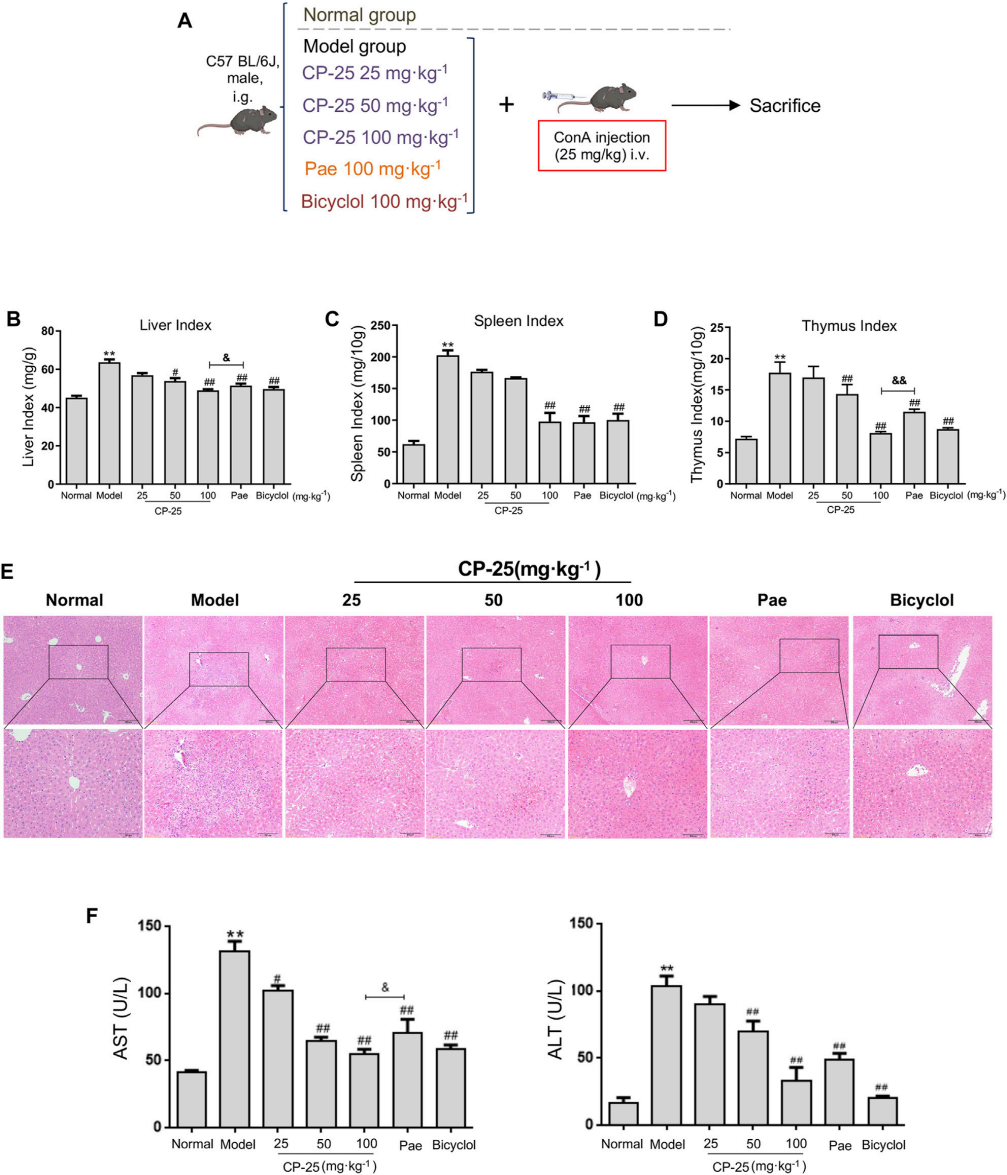
CP-25 attenuated ConA induced hepatitis. **(A)** ConA-induced hepatitis model and pretreatment of drugs. **(B)** Liver index. **(C)** Spleen index. **(D)** Thymus index. **(E)** The photomicrographs are of representative H&E-stained liver tissues after ConA injection of mice. **(F)** The serum AST and ALT activities. Data were representative as the means ± SD. **p < 0.01 *versus* normal, ^*#*^ *p* < 0.05, ^*##*^ *p* < 0.01 *versus* model, ^*&*^ *p* < 0.05, ^*&&*^ *p* < 0.01 *versus* paeoniflorin.

To investigate the effects of CP-25 on hepatitis, mice received an intragastric administration of CP-25 (25, 50 and 100 mg/kg) and intravenous tail injection of 25 mg/kg ConA (Figure 1A). Our results showed that the liver, spleen and thymus indices were increased post ConA injection. CP-25 (50, 100 mg/kg) gradually declined the above indices, and the liver and thymus indices in CP-25 100 mg/kg group were obviously reduced compared with the equal dose of paeoniflorin (Figures 1B–D). In addition, H&E staining observed visible histopathological changes of model mice, such as disorganized cell arrangement, necrosis with extensive inflammatory infiltration and hepatocyte nuclear lysis (Figure 1E), CP-25 treatment gradually alleviated the degree of liver injury. To further support histological analysis, the activities of AST and ALT were significantly elevated in liver injury mice (Figure 1F). CP-25 (50, 100 mg/kg) treated mice showed reduced AST and ALT activity. Additionally, CP-25 at 100 mg/kg obviously decreased AST activity as compared to the same dosage of paeoniflorin. These data preliminarily suggested a protective effect of CP-25 on ConA-induced hepatitis.

### CP-25 ameliorated T cells activation in hepatitis

**FIGURE 2:**
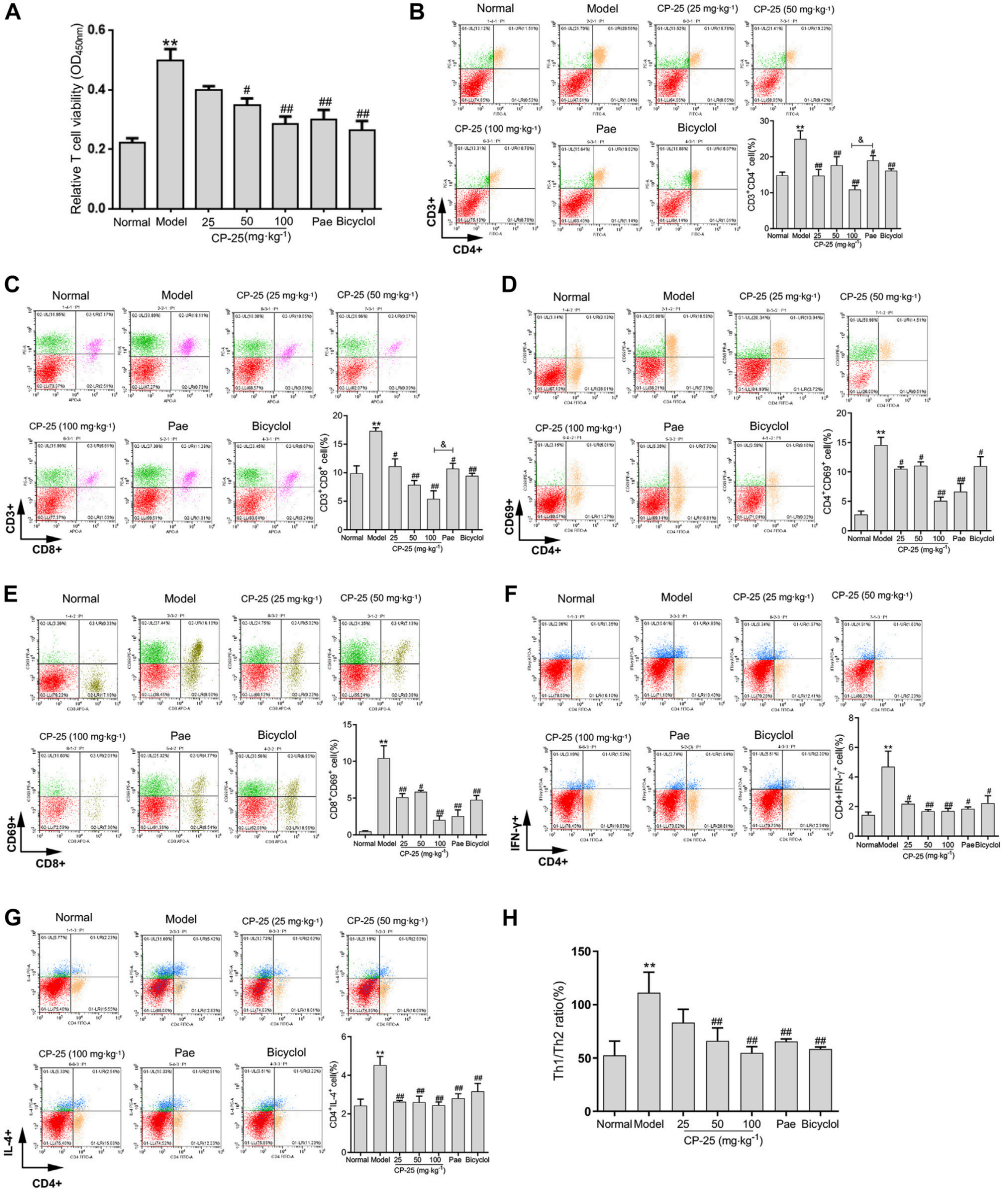
CP-25 ameliorated T cells activation in hepatitis. **(A)** The CCK-8 absorbance values of T cells from mice thymus. Flow cytometry analysis of single-cell suspensions of live immunocytes, which were isolated from freshly dissociated spleen of mice injected with ConA. The representative pictures and bar graphs showed the percentages of CD3^+^CD4^+^ T cell **(B)**, CD3^+^CD8^+^ T cell **(C)**, activated CD4^+^ T cell **(D)**, activated CD8^+^ T cell **(E)**, Th1 **(F)** and Th2 **(G)** cells. And the ratio of Th1/Th2 on each group **(H)**. Data of each marker were expressed as the mean ± SD at least three independent experiments. **p < 0.01 *versus* normal, ^*#*^ *p* < 0.05, ^*##*^ *p* < 0.01 *versus* model, ^*&*^ *p* < 0.05 *versus* paeoniflorin.

Inflammatory immune dysfunction supports the development of hepatitis (Zhao et al., 2020). Therefore, we used CCK-8 to test the T cell viability, which was markedly higher in ConA-injected mice than in normal mice (Figure 2A). But CP-25 (50, 100 mg/kg) treatment groups significantly reversed the changes of T cell viability in hepatitis.

T cell-mediated immune responses are thought to be involved in the process of ConA-induced hepatitis. To determine whether CP-25 altered T cell subsets in spleen, the proportion of CD3^+^CD4^+^, CD3^+^CD8^+^, activated CD4^+^ (CD4^+^CD69^+^), activated CD8^+^ (CD8^+^CD69^+^), CD4^+^IFN-γ^+^ (Th1 cells) and CD4^+^IL-4^+^ (Th2 cells) T cells were examined by flow cytometry (Figures 2B–H). The proportions of CD4^+^, CD8^+^, activated CD8^+^, activated CD4^+^ T cells and the ratio of Th1/Th2 were obviously elevated in hepatitis. In contrast, CP-25 treatment gradually down-regulated the proportions of CD4^+^, CD8^+^, activated CD4^+^, activated CD8^+^ T cells and the Th1/Th2 ratio. Moreover, the proportions of CD4^+^ and CD8^+^ T cells were significantly lower in CP-25 (100 mg/kg) administration than in the same dosage of paeoniflorin. These data suggested that CP-25 might attenuate T cell-mediated immune response through inhibiting the ratio of Th1/Th2 in hepatitis.

### CP-25 down-regulated the inflammatory cytokines release in ConA-induced hepatitis

**FIGURE 3:**
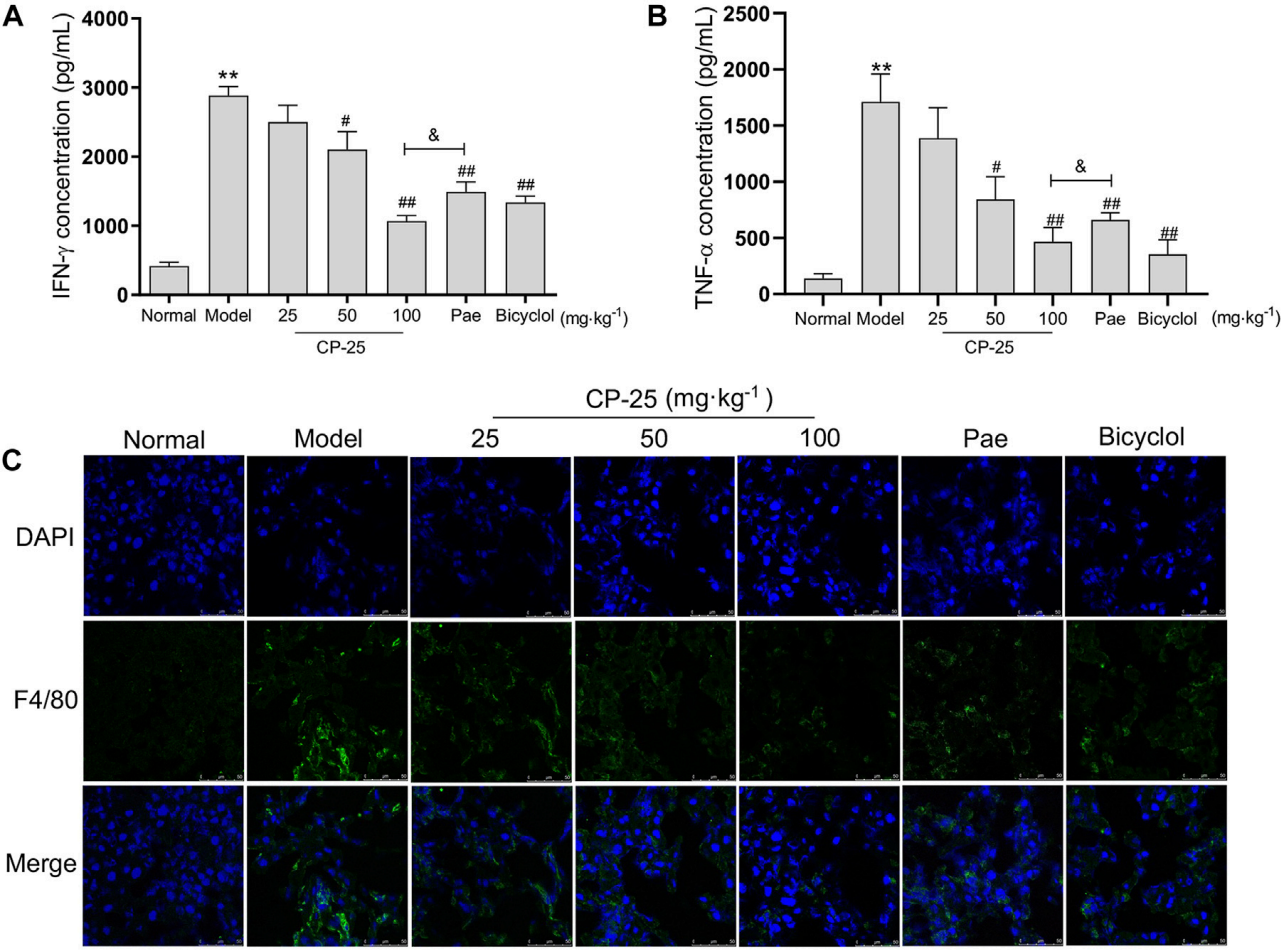
CP-25 down-regulated the inflammatory cytokines release in ConA-induced hepatitis. Comparison inflammatory factor expressions of IFN-γ **(A)** and TNF-α **(B)** in liver homogenate by ELISA. **(C)** Representative images showed the F4/80 positive staining cells (green) in liver sections by immunofluorescence (Scale bar = 50 μm). **p < 0.01 *versus* normal, ^*#*^ *p* < 0.05, ^*##*^ *p* < 0.01 *versus* model, ^*&*^ *p* < 0.05 *versus* paeoniflorin.

To further characterize CP-25 has a protective effect on inflammation in ConA-induced hepatitis, the production of IFN-γ and TNF-α were measured by ELISA (Figures 3A,B). ConA injection elevated the levels of IFN-γ and TNF-α, but CP-25 at dose of 50 and 100 mg/kg treatment suppressed the release of inflammatory cytokines. More importantly, mice given CP-25 (100 mg/kg) by oral gavage displayed lower levels of TNF-α and IFN-γ than mice treated with the equal dose of paeoniflorin. Due to macrophages are major sources of inflammatory cytokines during liver injury, hence we next analyzed the accumulation of macrophages (F4/80) in liver tissues by immunofluorescence staining (Figure 3C). Immunofluorescence staining showed an increase of F4/80 in ConA-injected mice compared to normal mice. However, CP-25 treatment suppressed ConA-induced F4/80 accumulation. Based on the above results, CP-25 might alleviate ConA-induced hepatitis via suppressing inflammatory cytokines release.

### CP-25 inhibited the activation of MAPK pathway and nuclear translocation of NF-κB mediated by ROS *in vivo*

**FIGURE 4:**
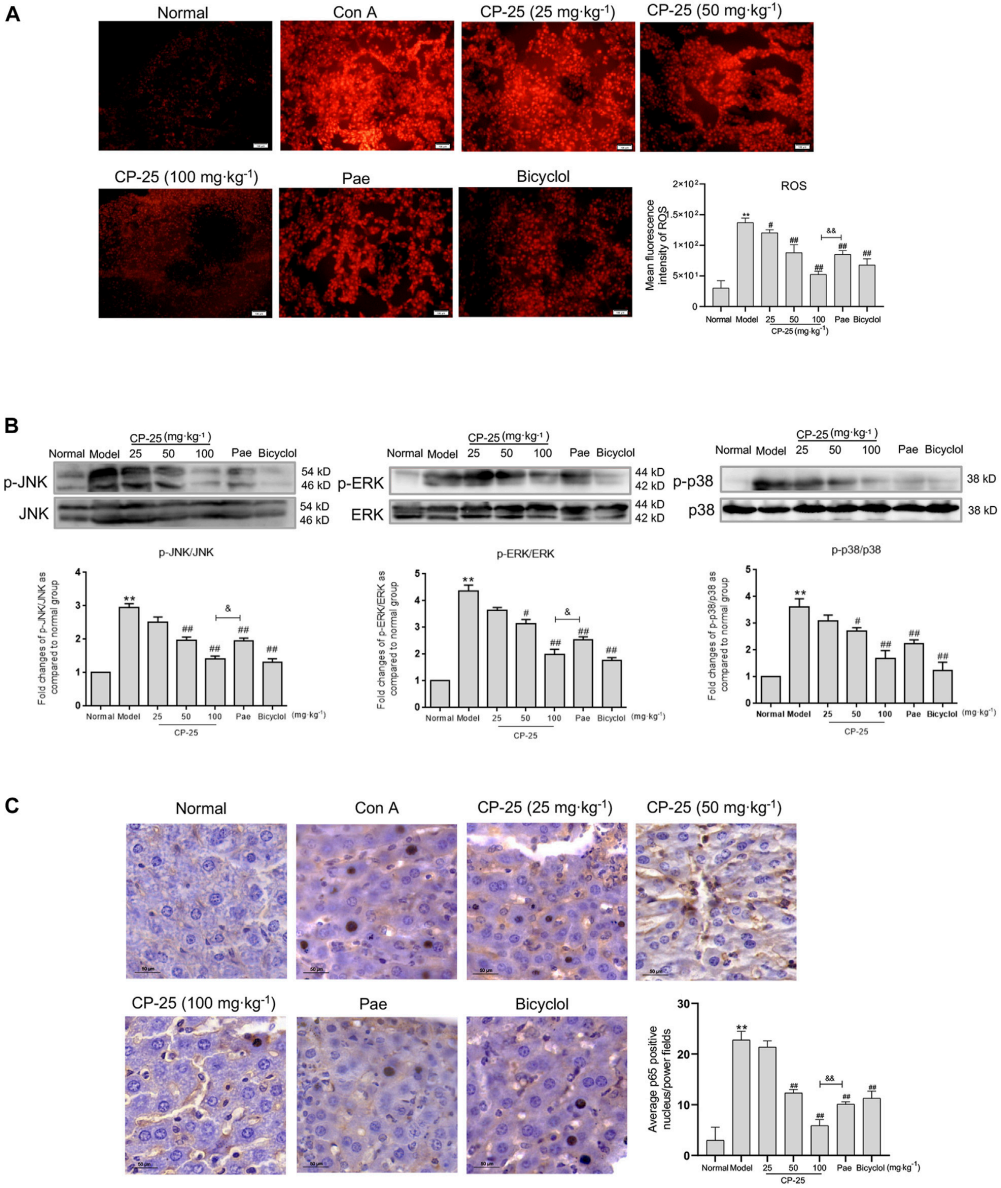
CP-25 inhibited the activation of MAPK pathway and nuclear translocation of NF-κB mediated by ROS *in vivo*. **(A)** Representative micrographs of ROS production in liver tissues were stained with DHE (Scale bar = 100 μm), and was quantitated by measuring the fluorescence intensity. **(B)** Representative immunoblotting bands of p-JNK, p-ERK and p-p38 in ConA-injected mice were showed, the semi-quantitative analysis was shown as the bar diagram. Densitometry values were expressed as-fold change relative to the normal group. **(C)** Representative images of NF-κB nuclear translocation in liver tissues were detected by immunohistochemistry staining (Scale bar = 50 μm). Results from three independent experiments are reported as the means ± SD. **p < 0.01 *versus* normal, ^*#*^ *p* < 0.05, ^*##*^ *p* < 0.01 *versus* model, ^*&*^ *p* < 0.05, ^*&&*^ *p* < 0.01 *versus* paeoniflorin.

Since ROS acts as secondary messenger affecting macrophage activation leading to inflammatory cytokines secretion (Du et al., 2020), the results of ROS level in liver tissues were showed in Figure 4A. ROS level was significantly upregulated in livers of ConA-injected mice, but CP-25 treatment downregulated the increased level of ROS. Researches have demonstrated that ROS induced the phosphorylation of downstream MAPK pathway (Gong et al., 2019; Wu et al., 2021). Additionally, NF-κB has been proposed to be the sensor for oxidative stress that can be activated by ROS (Morgan and Liu, 2011). When exogenous inducers were applied into cells, NF-κB dissociated from the cytoplasmic complex and translocated to the nucleus, which contributed to inflammatory cytokines production (Giustarini et al., 2020). Therefore, we detected MAPK activation and NF-κB nuclear colocalization in liver tissues. ConA-stimulated group exhibited that upregulated phosphorylation of MAPK pathways and increased nuclear translocation of NF-κB, but CP-25 treatment suppressed MAPK pathway activation and NF-κB nuclear translocation (Figures 4B,C). In addition, the highest dosage of CP-25 could obviously disrupt the levels of ROS, p-JNK, p-ERK and NF-κB nuclear translocation compared to the same dosage of paeoniflorin. These data indicated that CP-25 suppressed inflammation of hepatitis may be associated with down-regulating the MAPK activation and NF-κB nuclear translocation influenced by ROS.

### CP-25 inhibited inflammatory cytokines and ROS release in RAW264.7 cells

**FIGURE 5:**
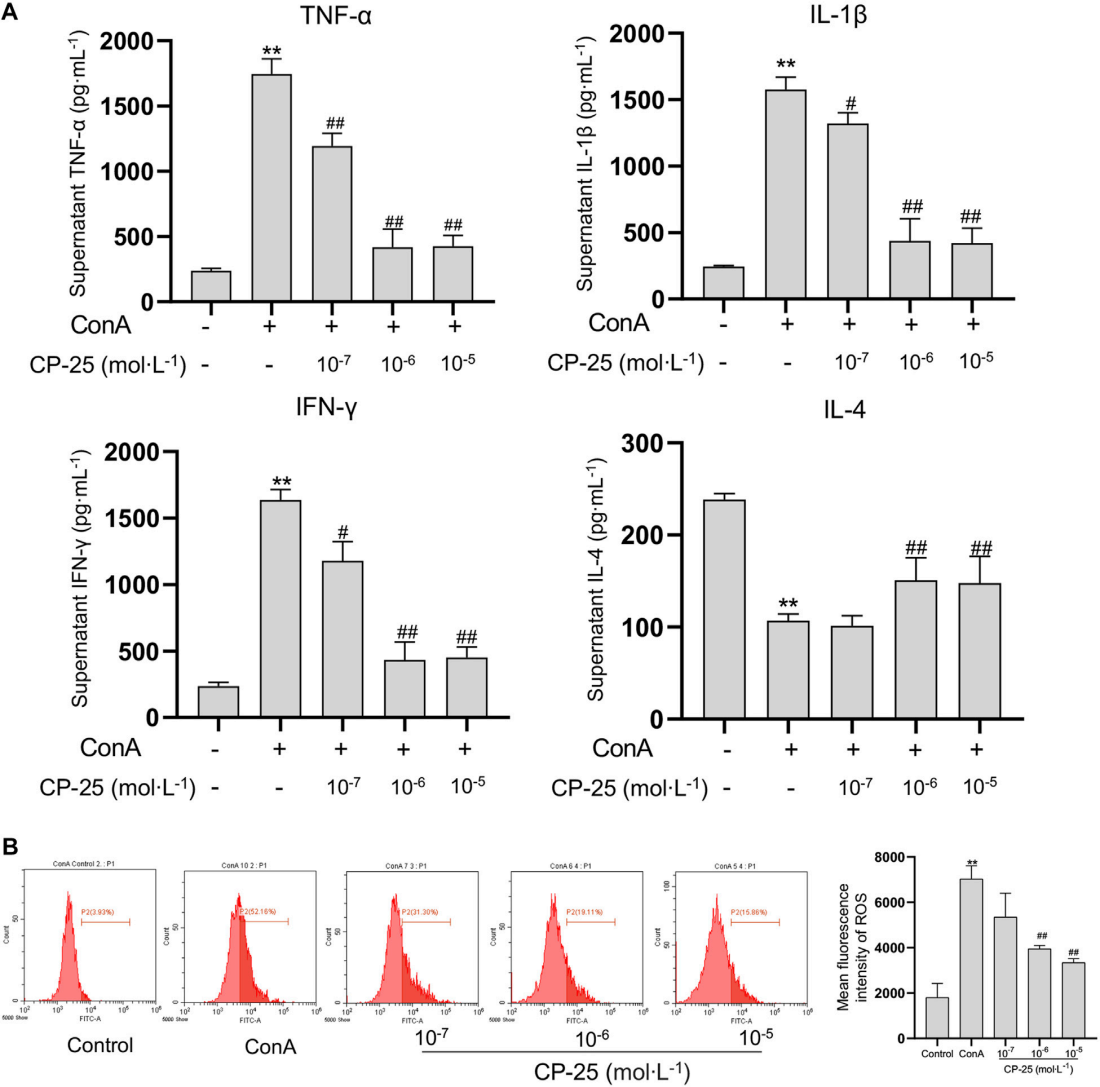
CP-25 inhibited inflammatory cytokines and ROS release in RAW264.7 cells. **(A)** Comparison inflammatory cytokine levels of TNF-α, IL-1β, IFN-γ and IL-4 in the supernatant of RAW264.7 cells by ELISA. **(B)** Decreased ROS levels were observed in CP-25 administrated RAW264.7 cells. **p < 0.01 *versus* control, ^*#*^ *p* < 0.05, ^*##*^ *p* < 0.01 *versus* ConA. Data were showed as the mean ± SD of three independent experiments.

Due to macrophages play an essential role in initiating inflammation, we utilized RAW264.7 cells *in vitro* to evaluate the anti-inflammatory effect of CP-25 (Wang et al., 2019). The levels of cytokines including IL-1β, TNF-α, IL-4 and IFN-γ were examined to verify whether CP-25 regulated inflammatory cytokines production *in vitro*. Results showed that ConA stimulation upregulated IL-1β, TNF-α and IFN-γ production, but reduced IL-4 release in RAW264.7 cells as compared to the untreated cells (Figure 5A). Furthermore, CP-25 treatment reversed the changes of inflammatory cytokines levels caused by ConA. Research showed that ROS as an effective molecule for determining the extent of inflammation (Ma et al., 2018). Hence the DCFH-DA fluorescent probe was used to detect ROS formation in ConA-stimulated RAW264.7 cells. The results showed that ConA stimulation significantly increased ROS production compared to the controls, while CP-25 (10^−6^–10^−5^ mol/L) ameliorated ROS production (Figure 5B). These data showed that CP-25 may inhibit inflammatory cytokines secretion accompanied with down-regulating ROS production in RAW264.7 cells.

### CP-25 suppressed ROS influenced MAPK pathway activation and NF-κB nuclear translocation in RAW264.7 cells

**FIGURE 6:**
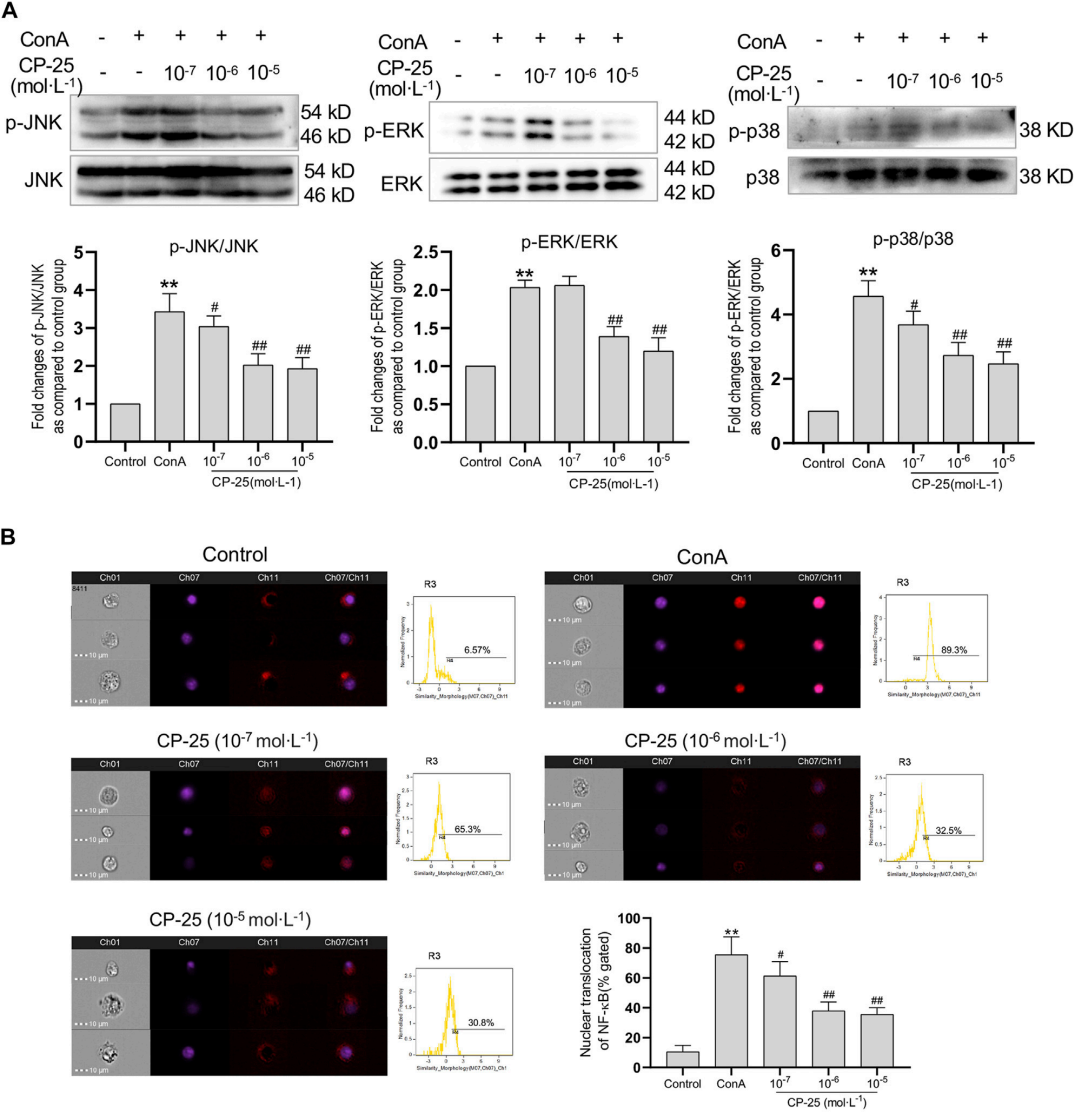
CP-25 suppressed ROS influenced MAPK pathway activation and NF-κB nuclear translocation in RAW264.7 cells. **(A)** Representative immunoblotting bands of p-JNK, p-ERK and p-p38 in ConA-stimulated RAW264.7 cells. Data were expressed as-fold change relative to the control group, which was assigned as a value of 1. **(B)** NF-κB nuclear colocalization in CP-25-administrated RAW264.7 cell was analyzed by image flow cytometry, and representative images were shown including brightfield, NF-κB (red), nucleus (purple), and merged images (Scale bars = 10 μm), and statistic results were shown. **p < 0.01 *versus* control, ^*#*^ *p* < 0.05, ^*##*^ *p* < 0.01 *versus* ConA. Data were expressed as the mean ± SD from three independent experiments.

To further examine whether CP-25 has an effect on ConA-induced MAPK activation and NF-κB nuclear translocation *in vitro*. The activation of MAPK pathway was detected by Western blot and the nuclear localization of NF-κB was examined by imaging flow cytometry. Results showed that ConA stimulation significantly up-regulated phosphorylation of MAPK pathways and nuclear localization of NF-κB compared to the controls (Figures 6A,B). CP-25 supplementation reduced the phosphorylation of MAPK signaling pathways and the proportion of NF-κB nuclear localization, which suggested that CP-25 could suppress inflammation may be through inhibiting MAPK activation and NF-κB nuclear localization in macrophages.

## Discussion

Hepatitis has been risen worldwide mainly due to exogenous substances (drug misuse, alcohol abuse, toxins), disease or external stimuli (viral infection, COVID-19), and others, which threatened human health recent decades (Liu et al., 2022). ConA-induced hepatitis was first established in 1992, which is now the most widely used tool to reveal liver injury in mice (Tiegs et al., 1992). Advanced evidence confirmed that various degrees of oxidative stress, inflammation, and necrosis could impair tissue function and integrity in ConA-treated mice (Khan et al., 2021). ConA altered cellular metabolic processes, which was contributed to disrupting oxidative phosphorylation and aggravating mitochondrial damage. Exhausted energy storage and accelerated production of ROS would cause damage to macrophages (Zanluqui et al., 2020). In this study, hepatitis model was established by intravenous injection of ConA and to explore the potential effect of CP-25. Pretreatment of CP-25 (100 mg/kg) exerted obviously hepatoprotective effects compared to the equal dose of paeoniflorin, includes reduced necrotic areas, decreased inflammatory cell infiltration.

CP-25, a novel ester from structural modification of paeoniflorin, has been shown to obviously anti-inflammatory activity in various diseases. Both in collagen and adjuvant-induced arthritis, CP-25 exhibited powerful anti-inflammatory and immunoregulatory effects and attenuated synovium inflammation (Chen et al., 2018; Wang et al., 2018). Furthermore, CP-25 alleviated kidney injuries in rats with arthritis through reducing the number of renal CD68^+^ cells and downregulating the levels of TNF-α and IL-6 (Wang et al., 2020). As we all known CD4^+^ cells are T helper cells which could differentiate into several subpopulations, and the activated CD8^+^ T cell subsets generally perform cytotoxic T cell functions (Basu et al., 2021). Research determined that the upregulated ratio of CD4^+^/CD8^+^ occurred the pivotal contributing factor in autoimmune diseases, virus infections and cancers. It was obvious that bulk CD4^+^ T cells differentiated into Th1 and Th2 subsets during autoimmune diseases progression (Zolfaghari et al., 2021). Th1 cells secreted Th1-type cytokines, such as IFN-γ, which positively fed back to promote further T cell differentiation and proliferation, and were associated to the cellular immunity response. Th2 cells could produce IL-4, which mainly participated in the humoral immunity in pathogenesis of autoimmune diseases. The dysregulation of Th1/Th2 would contribute to immunologic disease, such as osteoarthritis, hepatitis, type-1 diabetes and COVID-19 pandemic. Additionally, CP-25 reduced the infiltration of Th1/Th2 cells, and reduced inflammatory cytokines production in autoantigen-induced Sjögren’s syndrome mice (Gu et al., 2018). Then, we paid more attention on the relationship between CP-25 and T cell activation in ConA-induced hepatitis. The results confirmed that CP-25 treated mice performed a lower proportion of activated CD4^+^, CD8^+^ T lymphocytes and the ratio of Th1/Th2 than in ConA-injected mice. Furthermore, the highest dosage of CP-25 significantly reduced the proportion of CD4^+^ and CD8^+^ T cells compared with the same dosage of paeoniflorin group. These results indicated that CP-25 attenuated ConA-induced hepatitis may through modulating immune responses.

It is well-known that pro-inflammatory cytokines secretion could amplify inflammatory responses in immune cells (Feng et al., 2021). Our present findings occurred that CP-25 reduced inflammatory cytokines production and ROS levels in ConA-induced hepatitis. A sensible reduction in inflammation and ROS production was determined in CP-25 (100 mg/kg) treated mice compared with the equal dose of paeoniflorin. Paeoniflorin has been reported that inhibited TNF-α expression in BCG plus LPS induced liver injury (Liu et al., 2006). And other study revealed that CP-25 concentration was higher in liver tissue compared to paeoniflorin due to its elevated oral bioavailability (Zhao et al., 2019). Research supported that there was a positive correlation between ROS elevation and inflammation related pathways activation (Brennan and Gilmore, 2018; Yunrong Yang et al., 2022). And oxidative stress was happened accompanied by excessive ROS levels, which acting as signaling molecules contributing to abnormal cell growth, metastasis, disordered function, as well as enlarged proinflammatory cytokines production, allowing inflammation to progress (Li et al., 2020). Research showed that excessive ROS production may contribute to M1-like pro-inflammatory macrophages during the development of diabetes (Wada et al., 2017; Rendra et al., 2019). CP-25 regulated macrophage polarization from a M1 to a M2 phenotype to attenuate DSS-induced colitis and inhibited IL-1β and IL-18 production in mice (Li et al., 2021). As expected, our study determined that F4/80 positive staining was enhanced in hepatitis and decreased after CP-25 administration. Meanwhile, CP-25 treatment inhibited ROS production accompanied with down-regulation of inflammatory cytokines. Therefore, we speculated that CP-25 may protect hepatitis through prevention of ROS-mediated inflammatory cytokines released.

Then we identified the underlying mechanisms related to anti-inflammatory effects of CP-25 in hepatitis. The activation of MAPK was involved in macrophages accumulation, release of inflammatory factors and chemokine expression, which was involved in regulating tissue inflammation (Zhang et al., 2017). Studies presented that Zeaxanthin could induce a mass apoptosis of gastric cancer cells following by activation of ROS-mediated MAPK and NF-κB signaling pathways (Sheng et al., 2020). Another research demonstrated klotho downregulated inflammatory responses through inactivation of ROS/p38 MAPK pathways, leading to alleviate paraquat-induced lung injury (Zhang P et al., 2020). In present experiment, we demonstrated that CP-25 decreased the activation of MAPK signaling pathways. Besides there was compelling evidence to suggest that NF-κB could be activated by various stimuli including excessive ROS. H_2_O_2_ could rapidly activate NF-κB, which participated in ROS-induced cell death (Lingappan, 2018; Tian et al., 2021). It has been reported that sub-anesthetic ISO post-conditioning decreased the inflammation in OGD-insulted microglia partly *via* blocking ROS-NF-κB signaling pathways (Yao et al., 2020). Moreover, the NF-κB molecules were usually retained in cytoplasm in untreated cells. Upon stimulation, NF-κB p65 was able to migrate from the cytoplasm into the nucleus (Zinatizadeh et al., 2021). NF-κB p65 translocation promoted alveolar hypercoagulation and fibrinolysis in LPS-induced acute respiratory distress syndrome mice (Wu et al., 2020). NF-κB p65 has the tendency of translocation towards nucleus in LPS-induced BV2 cells to support the transcription of downstream pro-inflammatory factors such as IL-1β (Chen et al., 2022). Once activated by stimulators, NF-κB translocated into nucleus and involved in pro-inflammatory cytokines production (Luo et al., 2022). And in our results, CP-25 inhibited NF-κB nuclear translocation *in vivo* and *in vitro*. We supposed that the protective mechanisms of CP-25 against hepatitis had a direct relation to shut down ROS influenced MAPK activation and NF-κB nuclear translocation.

## Conclusion

In summary, our present data suggested that the protective effects of CP-25 administration on ConA-induced hepatitis through inhibiting immune response and inflammatory cytokines production. The underlying mechanism of CP-25 may relate to regulate ROS influenced MAPK activation and NF-κB nuclear translocation. Our studies contribute to providing new insights into the pathogenesis of hepatitis, and future research will focus on CP-25 as a potential therapeutic candidate for the treatment of hepatitis.

## Data Availability

The original contributions presented in the study are included in the article/supplementary material, further inquiries can be directed to the corresponding authors.
